# Older Adults’ Perceptions of the Ageing Experience in Trinidad and Tobago: A Qualitative Study

**DOI:** 10.1007/s10823-026-09592-x

**Published:** 2026-07-22

**Authors:** Sasha Bernard, Diadrey-Anne Sealy, W. Lawrence Beeson, Naomi Modeste

**Affiliations:** School of Public Health, Loma Linda University, Loma Linda, CA 92350 USA

**Keywords:** Ageing, Perceptions, Qualitative, Gerontology, Trinidad and Tobago

## Abstract

This study explores older adults’ perceptions of the ageing experience in Trinidad and Tobago, and gains insight into their self-perceptions of ageing. A concurrent mixed-methods approach was utilized, targeting older adults living in Trinidad or Tobago. For this paper, only the qualitative component of the study will be discussed. Thirty-eight (38) participants engaged in **six** focus group discussions about their physical, mental, and social health and ageing experiences. The focus groups were audio-recorded and transcribed verbatim. The data were analyzed with MAXQDA software and themes found. Participants lived experiences were garnered from various aspects of their lives, including personal matters and cultural and societal influences with major themes such as ageism and disrespect, undesirable cultural norms, and fear of crime. Most themes reflected positive perceptions, including positive outlook on ageing, positive perceptions of one’s health, enjoying retirement, satisfaction with available resources, and spirituality/religion. Negative experiences focused on the health system and in navigating financial challenges. The findings of our study show the need for a change in the framing of age and ageing at both the individual and societal level and shows the relevance of continued societal participation and adequate health care among older adults for a healthy ageing society. Multilevel and multisector efforts are required to reduce everyday ageism and promote positive beliefs, practices, and policies related to aging and older adults.

## Introduction

One of the defining global trends of our time is population ageing. Global statistics show that in 2019, there were one billion persons aged 60 years and older. It is estimated that by the year 2030, this number will increase to 1.4 billion and then by 2050, to 2.1 billion (World Health Organization, 2022). The world’s population of older adults is increasing by both number and proportion as people are living longer. The Decade of Healthy Ageing has been identified by the United Nations (UN) as 2020–2030 (Keating, 2022). Within the Caribbean region, history was made at the beginning of the 21st century with the population of persons 60 years and older being larger than ever before (Rawlins, 2008). According to the World Population Prospects the largest number of countries and areas among those that are likely to see the size of their population peak over the course of the next 30 years are in Latin America and the Caribbean (United Nations, 2024).

Trinidad and Tobago is a twin-island Caribbean nation, and one of the largest Caribbean islands located in the southern Caribbean Sea, with a rich history and culture. Over the past five decades, Trinidad and Tobago has seen a marked increase in its population of older persons (MPSDFS, 2024). Between 2000 and 2024, Trinidad and Tobago’s population grew from 1,319,774 to 1,507,782 people, representing an overall rise of 14.2%. During this period, the age structure also shifted as by 2024, adults aged 65 and older made up 12.4% of the population, which is 7.5% points higher than in the year 2000. (Pan American Health Organization, 2023). According to the World Bank, a country is considered as ‘ageing’ when more than 7% of its population is 65 years old and older (2021) .

The country is classified as a resource-rich, high-income country, with an economy driven by oil and gas production (Henry, 2021). Despite this classification, economic disparities persist, particularly affecting older adults who are one of the more vulnerable groups based on the fact that their income generation capacity is generally lower than those of working age. Many of these older adults often rely on limited fixed incomes such as pensions and grants however, the country’s non-contributory pension is subject to a means test, meaning its value is reduced for individuals who have other sources of income. Despite this, nearly two-thirds of people aged 65 and older still qualify to receive the non-contributory pension (Quashie & Jones, 2023). Trinidad’s healthcare system has two tiers: a government-funded public sector that delivers basic medical services to everyone, and a private sector that offers additional or higher-level care for those who can afford it. However, according to Bahall (2018) health system inequities in Trinidad still exist and they disproportionately burden the poor, who may rely more on costly out-of-pocket care, while the rich benefit from better access and informal networks. Social support systems are largely family-based, though these are increasingly strained due to changes in family structures and the associated economic pressures such as the outmigration of younger adults, many of whom leave in search of better economic opportunities and education, thereby reducing the traditional caregiving pool for the elderly (Moonesar et al., 2016).

Ageing, though it is considered a biological process, is commonly characterized by challenges, including the emergence of several complex health states (World Health Organization, 2022). While the process of ageing itself is not a disease, it increases one’s vulnerability to disease (Mahishale, 2015). For many reaching a certain age marks the beginning of diminished functional capacity and catalyzes several changes in the body such as greyed hair, wrinkled skin, loss of muscle and bone mass resulting in gradual reduction in height and weight, andropause in men and menopause in women. Sourtzi et al. (2019) noted that older adults encounter a range of physical, psychological, and social challenges that can affect their sense of identity and overall quality of life. It is also common in later life for individuals to experience one or more age-related physical health conditions. As life expectancy rises across the Caribbean, the burden of non-communicable diseases (NCDs) has increased in parallel. NCDs now account for more than 80% of all deaths in the region, with cardiovascular diseases, cancer, diabetes, and chronic respiratory conditions being the leading causes (Flores et al., 2025).

These age-related challenges are not limited to just physical health. Lee (2020) states that more than one in five older adults globally, experience some form of mental health condition. Common concerns in this age group include depression, anxiety, loneliness, and social isolation. Among these, depression is particularly prevalent, though its true rate may be underreported, as many older adults attribute symptoms to normal aging or other factors rather than recognizing them as signs of depression. Suicide is another significant age-related mental health issue, with a quarter of the deaths from suicide occurring globally among people aged 60 or older (World Health Organization, 2023). Many factors can lead to various physical and mental disorders in older adults. Health professionals and the broader public increasingly acknowledge social isolation and loneliness as significant public health challenges in high-income countries as both social isolation and loneliness are associated with an elevated risk of mortality. Nearly one in four community-dwelling older adults experience social isolation. (Blazer, 2020). This has been a concern even before the COVID-19 pandemic that started in 2019. However, with older adults being the demographic group who experienced social isolation for the longest duration during that period, the related health issues within that population have since been exacerbated.

Despite the diversity in ageing, a number of challenges are considered to be common, including declining quality of health, loneliness, impoverishment and social isolation (Pakulski, 2016). (Fike, 2018) highlighted common areas that older adults worldwide experience challenges in such as engagement and purpose, financial wellness, mobility and movement, daily living and lifestyle, caregiving, care coordination, brain health, and end of life.

The complexity of comprehending ageing, the diversity seen in older age, the nature of age-related problems, and the social and socio-economic impact of the inevitable process, all pose challenges and opportunities as it relates to the care, management, maintenance, and well-being of the older persons. These demographic and epidemiological trends suggest increasing pressure on both social and health systems to address the needs of a growing ageing population. According to the World Health Organization (WHO), a major challenge that all countries face, or will face, is ensuring that their health and social systems are ready to make the most of this demographic shift (World Health Organization, 2022). They must find effective strategies to extend care and to respond to the needs of this population. Public health professionals are also challenged to provide a comprehensive public health response to address the wide variety of older people’s experiences and needs. With the significant change in the global population as it relates to ageing, this study can provide insight on the adaptations required on the way societies are structured across all sectors as we work towards the Healthy People 2030 older adults’ goal to improve health and well-being for older adults (Office of Disease Prevention and Health & Promotion, 2020).

This study was guided by the General Ecological Model of Ageing and the Active Ageing Framework. The General Ecological Model provided a multidimensional lens to examine how individual, social, environmental, and policy-level factors interact to shape the ageing experience (Satariano & Maus, 2017). Complementing this, the Active Ageing Framework from the World Health Organization emphasized the importance of optimizing health, participation, and security in order to enhance quality of life as people age (World Health Organization, 2000/2002).

Understanding how older adults perceive, accept, and comprehend the ageing experience and process will allow for its nuanced challenges to be dealt with in an informed manner and provide critical insight in responsive policy and programmatic interventions to ensure healthy ageing and optimum quality of life for older adults. Gaining a better understanding of the individual perceptions of these older adults also allows the opportunity to ensure that their environment provides the necessary resources to improve and maintain the quality of their overall well-being. There is a dearth in the literature on older adults’ perception of ageing in Trinidad and Tobago. The purpose of this study was to understand older adults’ perceptions of the ageing experience in Trinidad and Tobago; and to gain insight into their self-perceptions of ageing.

## Methods

The study utilized a concurrent mixed-methods approach with a convergent design, however for the purpose of this paper, only the qualitative data will be presented and discussed. The relevant ethical committees approved this study; IRB #5,230,519 and He:3/13/441 Vol. II. The qualitative and quantitative data were collected in January and February 2024. Qualitative data were collected from Trinidadian and Tobagonian adults ages 60–85 (*N* = 38) through six focus groups to understand their perceptions of ageing and the ageing experience.

### Participants and Recruitment

Participants of this study were from a diverse group of adults ages 60 to 85, who were fluent speakers of the English language and residents of Trinidad or Tobago. The exclusion criteria excluded those who did not speak English fluently and those who exhibited observed poor coherence in thought and speech (e.g., those exhibiting signs of dementia). Focus group participants were recruited through advertisements posted on various social media platforms such as WhatsApp, Facebook, Instagram, LinkedIn, and Twitter. Recruitment was also done through an article published in a local newspaper, snowball sampling (Johnson, 2014) and through verbal communication at clinics and other public spaces frequented by older adults.

### Data Collection

Focus groups were selected as the qualitative research method to facilitate an in-depth understanding of participants’ perceptions and experiences in a social context. A total of six (6) focus groups were conducted at communal locations in major cities (*N* = 5) and a senior citizen residential home (*N* = 1). Thirty-eight (38) persons participated in these 6 focus groups; a breakdown of each group’s participants can be seen in Table 1. The focus group numbers were intentionally small to ensure that the participants would be able to provide in depth answers and to foster open discussion, while still reaching saturation (Hennink & Kaiser, 2022).

The focus group discussions were moderated by the first author (SB) with trained volunteers acting as note takers. A semi-structured guide was used to generate discussion with 10 open-ended questions including:

1. “How do you feel about “getting old”?”
2. “What would you say are positive things about growing old in Trinidad and Tobago?”
3. “Do you have any concerns and/or challenges associated with getting older?”
4. “When these challenges arise, or any other occur, how do you deal with them?”
5. “In dealing with challenges, is there any one in particular that you lean on?”
6. “Do you feel that you have what it takes to ‘age gracefully and healthily’ in this country?”
7. “What are your views on the health services provided to you?”
8. “What are your views on the social services provided to you?”
9. “Is there anything in particular that you have put in place to ‘assist’ you in ageing?”
10. “What would an ideal experience of ageing in Trinidad and Tobago look like to you?”

The focus group questions were guided by the General Ecological Model of Ageing and the WHO Active Ageing Framework to ensure a well-rounded exploration of older adults’ self-perceptions of ageing in Trinidad and Tobago. The ecological model shaped questions that consider influences at the individual, interpersonal, community, and societal levels such as personal feelings, family support, community resources, and national services. The Active Ageing Framework further directed the questions toward understanding the key elements of health, participation, and security, allowing participants to reflect on their wellbeing, engagement, safety, and preparedness as they age. Together, these frameworks ensured that the questions captured a comprehensive, holistic exploration and understanding of both lived experiences and the wider cultural and structural factors that shape the ageing experience within the unique sociocultural context of Trinidad and Tobago.

Data were collected until data saturation was reached. Each of the focus groups lasted approximately 60 min and were audio-recorded and transcribed verbatim. The participants received a gift card valued at $7.00 USD ($50.00 TTD) and were provided with refreshments.

### Data Analysis

Data analysis was done in three (3) phases based on the convergent design that the study utilized. Phase 1 included data analysis of the qualitative data was done by coding the data and collapsing the codes into broad themes. After reviewing the transcript and observational notes, analysis of the qualitative data were done by the first author (SB). Intercoder reliability was achieved with the second author (DS) after the preliminary review. Thematic analysis of the qualitative data was done using MAXQDA Analytics Pro software (VERBI Software, Berlin, Germany). A deductive analysis was conducted on each question. Phase 2 included the analysis of the quantitative data, and the final phase involved the mixed-methods data analysis (i.e., integrating both qualitative and quantitative results in the study) through a process that is referred to as triangulation to enhance the validity and credibility of the findings. Only the qualitative results are discussed in this paper. The quantitative results will be discussed in a subsequent paper.

## Results

### Findings

Fifty (50) participants expressed interest and registered to be a part of the focus groups. Of those who expressed interest, 38 participants showed up to the specified venues and were a part of the six focus groups (see Table 1**)**. The thematic analysis showed common perceptions on various facets of the ageing experience.

**Table 1:** Demographic distribution of focus group participants (*n* = 38)

Variable	Frequency	%
Age group		
60–64	15	39
65–69	8	21
70–74	9	24
75–79	4	11
80 and over	2	5
Gender		
Female	33	87
Male	5	13
Region		
Region 1^a^	6	16
Region 2^b^	13	34
Region 3^c^	1	2
Region 4^d^	6	16
Region 5^e^	8	21
Region 6^f^	4	11

Data were received from ^a^ City in South Trinidad. ^b^ City in East Trinidad. ^c^ City in West Trinidad. ^d^ City in Central Trinidad ^e^ City in Tobago ^f^ Home in East Trinidad.

Thematic analysis of the data collected on how individuals’ perceptions of ageing shape or relate to their ageing experience revealed several interconnected themes that suggest a strong relationship between how individuals view ageing and how they navigate it.

Positive outlook on ageing, positive perceptions of one’s health, embracing, adapting to changes, and mental preparation for ageing were all highlighted in the analysis. The highlighted themes reflect key elements of both the General Ecological Model of Ageing and the WHO Active Ageing Framework (Satariano & Maus, 2017; World Health Organization, 2000/2002). They align with the individual level of the ecological model by highlighting older adults’ internal beliefs, coping strategies, and psychological resilience, while also suggesting supportive social and environmental contexts. Within the Active Ageing Framework, these themes demonstrate strong foundations in the pillars of health, participation, and security, as participants show confidence in their wellbeing, adaptability in staying engaged, and mental readiness for future changes.

The commonality of these themes underscore the importance of mindset and internal framing in influencing how ageing is experienced and perceived and illustrate how personal strengths and environmental supports shape a positive ageing experience.

### Positive Outlook on Ageing

Most participants had positive outlooks on ageing. Ageing was described as lovely, wonderful, exciting, a privilege and better than the alternative - death. One participant expressed how her views changed as she got older. “*When you were younger*,* you’d think old is meh…but old is wonderful.”.* Another participant explained why she felt good about ageing. *“You can get older in age but not in body*,* strength*,* and soul.”.* A lot of participants also expressed that they don’t feel “old”, nor do they feel their age. One male participant said “*I don’t feel as old. I think I am young. The fact that people even tell you - you’re looking so fresh.*“. Many female participants also echoed the sentiments of this participant who could not relate to feeling aged, “*I feel the same way as I did at 30 and 40*.” Gratitude was often present in participants speaking positively about their ageing experience. “S*uddenly I’m my parents’ age*,* here am I*,* you know? But I find joy*,* it is fun*,* and I am being thankful for good health and so on*,* that’s where I am*.”.

### Positive Perceptions of One’s Health

Most participants had no major physical or mental health concerns - nothing they were worried about. One participant, in reference to mental health said; “*your brain is functioning relatively normal*” while another, in reference to physical health said; “*I believe that I’m as strong and as healthy as I was a few years ago.*”. These participants generally had positive perceptions of ageing. However, those that did have health issues, expressed a negative outlook on ageing such as a participant who mentioned having heart problems in the recent past who said: “*I’m feeling as you get older*,* you feel like you’re getting more to the death mark*,* you know? Especially if you’re sick*,* you’re feeling like tomorrow or next week could be the day*,* because that’s how you feel.”.*

### Adapting to Modern Trends

While most participants embraced their current age and life stage, there were a few challenges in adapting to modern trends, such as technology. As one 80-year-old female participant said, “*One of my challenges*,* and it will seem very unambitious of me*,* is technology.*”. However, it was agreed upon by participants that they must adapt to remain relevant. Another participant who described herself as tech-savvy said, “*you were not always this age*,* and technology has been here for the past how many years*,* but people refuse to embrace it. So*,* if you had embraced it from before*,* using it now*,* is just nothing.* “.

One participant who was willing to become tech savvy hypothesized that even though it was challenging, with the right support it could be done. “*I think it’s something that the government needs to take the lead on*,* in terms of bringing the older population into the digital era*.”.

### Mental Preparation for Ageing

Preparing for ageing was also something that participants said made the process a bit easier. Male and female participants shared the same sentiment on this. One female participant said, “*I personally have always been preparing myself*” while one male participant said; “*I recognized long ago that this ageing is inevitable I prepared myself for it.*”. In retrospect, participants spoke of things that would have been done in their youth that affected them now as they are older. One participant elaborated; “*In your younger age you’d have forsaken a lot of things*,* but it’s habits that we were forming*,* and it affects us when we get older. Even though you might be saying*,* yes*,* you’re sprightly*,* you still must be cognizant of the fact that some things we did in our younger age would affect us now*.”. For them, it’s now a matter of unlearning bad habits and dealing with the consequences of decisions made in younger days.

A recommendation suggested by several participants, to ensure that ageing was a manageable and enjoyable experience for all, was to have pre-retirement seminars outlining what to expect from this phase of life. Most participants expressed that being educated on the subject would have been beneficial and that education should begin once you joined the working force rather than waiting until the time of retirement was near.

“*I think education has a lot to do with it -preparing for these occasions. We are at that stage now for funeral grants and other medical situations*,* we must make sure to put aside something for a rainy day. I don’t think that they should wait until five years before retirement to start the education. I think it should be as soon as people start working*.”.

The analysis of the question, “What are the lived experiences of older adults regarding the ageing experience in Trinidad and Tobago?” revealed an interplay of social, cultural, and personal factors that shape the daily realities of ageing in this context. Five key themes emerged from participants’ narratives. These were **ageism and **disrespect, undesirable cultural norms, fear of crime, enjoying retirement, and financial challenges. Collectively, these themes paint a nuanced picture of the ageing experience for participants, highlighting both challenges and moments of joy.

### Ageism and Disrespect

While a few male participants found that the respect they received in society had increased with age, most female participants spoke of experiences of disrespect and ageism in various situations. Some even experienced some level of invisibility in society because of their age and society’s perceptions of ageing and older adults, “*My greatest challenge*,* and I’ve given you the example that I recently had*,* is that I find people don’t see you*”. As a result, they have been treated with scant courtesy, “*When they see you old it seems like they put you aside*.”. Another participant reported that her levels of intellect have also been questioned because of her age, “*They look at old people*,* elderly people*,* as stupid people. Many of them*,* especially young people - because they feel that they would not get that age*,* you know?*”.

### Undesirable Cultural Norms

The cultural norms of how older adults are addressed was considered unfavorable by most female participants. For women, being called ‘tanty’ or ‘tants’ which is the local slang for older aunt, and for men, being called ‘uncle’. “*If you are going out somewhere they will say tanty or something like that*,* and then I realized*,* I don’t know if I always appreciate that*.”. It was acknowledged that the experience may not be the same for both genders, but it is received differently based on who the caller is. “*Sometimes I think some people mean it as a disrespect. Men may not experience it*,* but sometimes*,* I go into the market and this elderly lady is missing teeth and calling me tants*,* and sometimes you wonder*.”

### Enjoying Retirement

Retirement, for all participants, was a source of joy - the flexibility of free time, the opportunities to do things they could not do while working or not having to constantly be rushing. One participant who retired approximately two years ago talked about her engagement with leisure activities such as exercise. “*Now you can do things that you wouldn’t normally do. I am now in a cycle club*,* I swim*,* I do lots of things. I go to the beach every day if I can help it. Although I did some of those things while I was working*,* now it’s such a leisure*.”.

Another participant talked about accomplishing a long-held goal since being retired; “*I’m retired 10 years now and it’s very interesting that I was able to accomplish something that I wanted to do. In the first two years of my retirement*,* I wrote and published a book on wellness*.”.

Some participants acknowledged that their time had not been freed up as they were still involved in so many activities. “*I’m not feeling retirement yet because I’m involved in a number of NGOs*,* and they keep me busy. I just keep going and I have two dogs that I love dearly that I got close to retirement*,* so they’ve brought a new love to my life. Looking forward to the rest of it*.”

Participants also spoke about the sense of fulfillment experienced when meeting people who they either worked with or assisted at some point. One retired teacher explained, “*You had your job*,* you do what you have to do well*,* so well that people you meet later in life*,* they remember you. So that plays a part in helping now*.”.

### Financial Challenges

Retirement provided a change in pace but also resulted in a change in income. The adjustment from receiving a salary to now receiving a pension was a challenge experienced by most, especially as it related to preparing for the future, maintaining a home, eating and living healthily, and medical challenges. “*I would say for me*,* yes*,* the financial challenges are always there. I’m thinking about how I can make my little bit of money stretch*.”. Participants also spoke about the time taken to receive compensation; “*when you retire*,* there’s all these challenges to start to reap the benefits of your investment*.”.

One of the main concerns of having financial challenges was loss of independence. A male participant suggested that financial literacy courses are good strategies for older adults to learn to maintain financial independence, “*I’m talking about teaching them how to deal with pension*”.

Participants expressed the strong desire to not have to rely on anyone, not even family, for anything and thought preplanning was another way to maintain independence. “*You want to stay in a position that you’ll be able to do for yourself and not be dependent on anybody. You want to be independent. Then you will have put something in place while you’re working and say this is what I’m going to use when I’m retired*.”.

### Fear of Crime

The fear of crime, particularly those targeted towards older adults, was a barrier to fully enjoying the ageing experience, as expressed by numerous participants. One male participant, who was a victim of a robbery along with his wife, described the population as easy prey to criminals. The crime situation not only affected their public socialization but was also a source of worry as it related to their loved ones such as children and grandchildren.

A participant in Tobago shared her experience as a grandmother. “*If my granddaughter is going out at night*,* I don’t know that I would fall asleep*,* because you’re always wondering*,* “When is that car driving in the garage?*” *You worry because of what is out there. I didn’t have those thoughts when I was a young lady growing up. I am going and lime (hang out) with my friends freely. You didn’t have to worry about a gunman on a corner or somebody going to rape you somewhere.”.*

Despite the headlines in local newspapers and acknowledge from government ministers on the issue, participants wished that the government paid more attention to crimes targeted to this vulnerable population. A female participant in Trinidad shared; “*I am somewhat concerned when I have to come out of my house with the crime situation- when I’m home I say I need to double and triple lock. It is somewhat disconcerting*,* and things are not in place*,* things are not being done*,* but just mentally*,* it’s a little unnerving at times.”*.

The question, “What are their perceptions of the local health and social services regarding the ageing experience?” revealed themes which proposed a blend of both positive and negative sentiments among participants. Themes of satisfaction with available resources and positive perceptions of services offered coexisted with negative experiences within the systems. This dual reality highlights the difference in infrastructure and intent and the actual delivery of services, leading to mixed experiences in how participants perceive the systems meant to support them.

### Satisfaction with Available Resources

Overall, participants were satisfied with the local health and social services provided to them. Some of the commonly mentioned resources included free healthcare in the public sector, the free bus pass for those aged 65 years and over that enabled them to travel for free, monthly senior citizens’ pension for those over 65 years (government allocated grant); the reduced cost for air and sea travel, and the waiver of fees for renewal of driver’s permit and passport for those 60 years and over. The grants and programs offered by the Ministry of Social Development and Family Services to meet the needs of persons with inadequate resources, such as the public assistance grant and pharmaceutical grant.

Non-governmental organizations such as Trinidad and Tobago Association for Retired Persons (TTARP) which provided retail discounts were also frequently mentioned. “*Officially Trinidad & Tobago has a lot*,* the Ministry of Social Development has the Division of Ageing*,* the Ministry of Youth Development and National Service has the program with the youth*,* so officially there is*,* then you have agencies like TTARP. I think that there are efforts*,* compared to other Caribbean countries we are fortunate*,* officially at least*.”.

### Positive Perceptions of Services Offered

One participant stated, “*I think we have a pretty good health system as well*.” which was agreed upon by the majority. In sharing her experience about the local health services at a hospital in East Trinidad, one participant who had been in and out of hospital with health challenges within the past few years found that while the services were satisfactory, the persons providing the services left much to be desired.

“*I don’t have a problem with the facilities at the hospital. They’re very good. The hospital is an excellent hospital. They have all the facilities they have everything, but the nurses and the orderlies, no. They’re awful.*”.

It was acknowledged that at each visit you could have a different experience. “*I think it goes and comes*,* because there are times people complain of the situation they encounter*,* but there are times you get very good attendants too.*”. The negative experiences with the publicly available health care services however, outweighed the positive.

As it relates to the social services offered, participants appreciated the vast array of services offered. However, they agreed that the systems to access these services needed improvement. Two of the main issues highlighted in the discussions were the inconsistency of the more popular services such as Geriatric Adolescent Partnership Program (GAPP), which is a governmental program that provides home caregiving services for the elderly and the fact that people are not aware of a lot of the services/grants available.

### Negative Experiences Within the Systems

Many participants spoke of negative experiences at the hands of healthcare providers and hospital staff. This was reinforced by a participant who was a retired member of the healthcare system. “*There’s a lot of ageist attitudes when it comes to pain. And I think that is part of our culture*,* but it is exacerbated in health*.”. The ageist attitude was manifested in a statement made by one participant who had gone to Accident and Emergency (Emergency Room) in Tobago. She said, “*once you are old*,* they don’t have time to worry about you too much*,* as though they don’t want to waste the medicine on an old person*”. Another gave an experience of a friend who was hospitalized with COVID-19 related complications; “*the doctor told her*,* well we’ll wait until your niece comes and I’ll speak to her*.” and she had to tell him, “*I have all my faculties*,* I don’t have dementia yet - talk to me.*“. One male participant wished that doctors were more direct in their communication; “*If as a patient*,* I go to the doctor or wherever*,* I want him to tell me exactly*,* this is how this medication is working*,* and this one is not working*.“. Whether it was based on preconceived notions of older adults, or lack of cultural sensitivity, participants alluded to the fact that a lot of the medical and nursing staff had certain misconceptions of ageing. A recommendation made by a participant who had previously worked in the healthcare system, was to have specific training for health personnel to reduce ageist bias.

“*I think that a lot must be done particularly in health*,* to train the health personnel to understand that they are not dealing with their mothers*,* they’re not dealing with their fathers and that being 70 or 75*,* you’re still a person -you still can make decisions. And when people in healthcare come from abroad*,* they should be also training them to acclimatize them*,* culturally*.”.

The negative attitudes shown towards older adults by those in customer service were also stated as a challenge. One participant, a retired supervisor of customer relations said; “*it aches my heart when I see how they relate to people*,* especially the senior folks.*”. A participant spoke of her experience at an office with a customer service representative who was rude to her as she was asking questions. She felt that this interaction was because of her age; “*I said*,* excuse me*,* when I looked in the mirror before I left home*,* I saw a human being*,* and I’m seeing a human being in front of me*,* so why are you behaving like a pig and treating me like a dog?*“.

While the participants in Trinidad did not indicate any limitations in accessing health services, participants in Tobago (the smaller of the two islands), expressed challenges in accessing services at the local health centers. One participant’s lamentation was “*you always have to be rushing to Accident & Emergency (Emergency Room) which is overcrowded at times and can’t help everybody because all the health centers are closed*.”. In discussing the private healthcare sector vs, the public healthcare, a participant stated, “*I have made a conscious decision to use the state health system*”, as she along with many others considered the financial implications and the high cost of receiving private care as it was noted “*the private bills could empty your bank*”. The wait times at public health facilities were also deemed as a two-fold issue. Participants found the wait time to be seen very long and uncomfortable, but also the wait time for treatment once it was determined as necessary. One participant who had received a shoulder replacement using the private healthcare sector explained; “*I asked the doctor*,* can I be part of the (public) clinic*,* and he said*,* sure*,* I can put you on the list*,* but you will not be treated before 2025.*”

Participants who at some point attempted to access the services described the processes as ‘too long’ and the systems as ‘archaic’ - with persons opting out to avoid that stress. One participant who decided not to apply for any of the grants offered explained; “*to follow up*,* it’s six months*,* and at the social office you have to be prepared when you go there with your puzzle and your work to sit and all these people there*,* it is just not goo*d.”. Another participant concluded that the issues within the system are based on poor inter-organizational communication; “*I think one hand doesn’t know what the other hand is doing*.”.

Additionally, the participants from all regions reported that some of these government offices, and even some banks, did not have public restroom facilities accessible, nor seating areas - which made waiting very uncomfortable for older adults.

In exploring the question, “What coping mechanisms do these older adults use to deal with the challenges associated with ageing?”, thematic analysis revealed three prominent strategies that participants rely on to navigate the ageing process. These multidimensional strategies used to confront and adapt to the realities of growing older were social support, maintaining physical and mental health, and spirituality/religion.

### Social Support

Participants agreed that having a solid support system, and socializing were crucial in dealing with age-related challenges. One participant expressed the importance of consistent socialization. “*Because no matter what you’re doing*,* when (older) adults are left alone and not being able to communicate*,* especially daily it affects them mentally*,* and puts them in a depressive mood. The more they interact with other people at this age it’s better*.”

Another, in imagining the state of her mental health without her support system stated emphatically; “*if I did not have a support system*,* I’ll stand up at the side of the road with a placard.*” – alluding to the helplessness she would feel. A support system of friends, whether newly formed or from the past, was also something that participants valued as well - whether it be to talk to, to go out with, or even to communicate via WhatsApp. One female participant said; “*so that is part of how I cope too*,* having this close group of friends*,* who understand me*,* and I understand them*.”. A male participant however said; “*having the support of friends you had in the past*,* so I’m enjoying it*”. Several participants identified family members such as spouses, siblings, children, grandchildren, in-laws, and nieces and nephews as their support system.

One participant expressed how she was able to rely on various members of her family. “*I have a family that is there for me. Once you have that familial support*,* it’s easy for me to say to my son -hey can you help me out? And he says yes. My grandson is only 10 but he promised that he’s going to take care of me*.”.

Another participant who is wheelchair bound and lives in a senior citizen residential home for the past year mentioned a particular nurse, who provided support for her “*anything that I want to know or want help with*,* she is always there for me*.”.

Participants acknowledged the importance and benefits of intergenerational socialization, “*even though we have all the experiences*,* every time you speak to a younger person*,* you’re still learning something*,* so you have to have an open mind*.”. However, they were also able to admit how challenging developing those relationships were sometimes and a conclusion drawn by a participant highlighted the need for mutual respect. “*Sometimes when we are getting negative treatment from young people*,* we have to look at ourselves and see how we are treating them*,* and it will make a difference.”*. One participant shared an idea that she had for an intergenerational skillset exchange to foster socialization and personal development of both generations.

“*You can share your skillset and work with the younger generation*,* and vice versa they might want to teach you something*,* a skill that they are also learning. Whether they are musical and they teach an older person to play the piano or to play guitar*,* or you’ll be teaching them something that you have already experienced - so I like the idea of that generation guide*,* exchange skillsets.”.*

### Maintaining Physical and Mental Health

Participants expressed the importance of keeping themselves as active and engaged as possible. They emphasized it was not just about making time for themselves to relax and do activities that they like, but keeping their minds stimulated and their muscles moving. One said, “*but we have to make the sacrifice*,* make some time for ourselves*,* otherwise we’re going to go craz*y.” For some, that looked like keeping their brains active by learning new things. One participant said, “*keeping your brain active as you get older whether you’re a young person or not is important. It’s vital.*”. Another said, “*I actively look for courses - whether gardening*,* cooking*,* whatever*,* and I sign up and I do something because my brain is amazing*.”. For some, they picked up hobbies like gardening to improve their well-being. “*I’m trying to do planting outside and I’m feeling better.*”

The engagement in physical activity or exercise was also a very common coping mechanism with participants describing a variety of forms of exercise. Some walked, some went to the gym to lift weights, some incorporated it into their daily routine, and some did classes like aerobics. One participant, who still worked part time as a doctor spoke about walking: “*30 minutes*,* 5 days a week. You will see the difference for your physical health*,* mental health*,* emotional health*,* and spiritual health*.”. Another participant said, “*I walk*,* bicycle*,* sometimes do aerobics and I have a treadmill. So*,* I’ve got at least four days at least*,* sometimes five and if I have to get something at the market*,* I put my sneakers on and I walk*.”.

### Spirituality/ Religion

All participants did not identify as religious, but a few were involved in church ministries and activities and leaned on their spirituality to deal with challenges. One female participant in Trinidad in speaking about what kept her most busy mentioned, “*so I love to do things like that too. It helps. And preach the gospel*,* I sing*,* I am involved in evangelism*,* and I also got involved with school evangelism.*”. Another male participant in Tobago spoke proudly of his involvement in various activities. “*I am heavily involved in church work*,* in all sorts of groups*,* doing all sorts of things.*”.

## Discussion

This research sought to understand how older adults perceive and understand the ageing experience; and to gain insight into older adults’ self-perceptions of ageing in Trinidad and Tobago. It also explored their lived experiences, their views on health and social services and their coping strategies. As the population of older adults grows, it becomes increasingly important to understand the factors that can help each individual age well and live longer (Tully-Wilson et al., 2021).

According to Benyamini and Burns (2020) perceptions of both age and health have been shown to independently predict future health and well-being. However, research has often focused on one of these factors in isolation. Studies on perceived age have indicated that its impact on longevity may be influenced by an individual’s perception of their own health. Our study found that participants generally had a good perception of ageing and positive perceptions on their health and wellness. This was supported by research done on self-perceptions of ageing which suggested that the way older adults perceive their ageing can have correlations to health and well-being (Hausknecht et al., 2019). Having good health and well-being seemed to provide older adults with a sense of comfort as it relates to ageing.

Results from our study found that disrespect, ageism and other forms of abuse were commonly reported as lived experiences of older adults in Trinidad and Tobago. This was seen in various aspects of their lives, personal, cultural, and seeking services. In 2010, a local article stated that age discrimination in the country was often overlooked for more outwardly obvious forms of discrimination such as gender and racial discrimination, although it carried the same negative personal, social and economic implications (Llanos, 2010). The impact of ageism on health and well-being has been established in numerous studies. Even within the United States, (Allen et al., 2022) found everyday ageism to be prevalent among older adults ages 50 to 80 years though the association with poorer health and well-being among older adults was only seen in major incidents of ageism. However, Kang and Kim (2022) found that older adults with a high level of psychological well-being, especially those who were proud of their age group, were less negatively affected by ageism, experienced less negative emotions, and were more optimistic about ageing and their future. Despite existing legislations such as Homes for Older Persons Act in Trinidad and Tobago, in 2023, the Minister of Social Development and Family Services reported that of the 216 reports of abuse elderly people to date for 2023, 182 of those cases were in private residences and 34 occurred in homes for the elderly (Khan, 2023).

The results also revealed negative experiences within systems. Some of the challenges within public systems, such as the healthcare system, could also be attributed to lack of preparedness, or lack of knowledge in dealing with an ageing population. A study done a few years ago reported that primary care physicians in Trinidad and Tobago expressed limited knowledge of clinic protocols related to elder abuse, low participation in training on recognizing and reporting abuse, and a tendency to take a hands-off approach to non-medical abuse issues (Huggins et al., 2021). Another study (Rawlins et al., 2015) emphasized the importance of undergraduate pre-healthcare students, future health professionals, acquiring higher levels of knowledge and increased understanding of Alzheimer’s disease and other-related dementia illnesses to prepare them to meet the challenges of caring for the increasing number of cognitively impaired patients as the world’s population ages.

Ageism is expressed through cultural values and social beliefs, as with other types of discrimination. Trinidad and Tobago consists of many diverse cultural groups and Bergeron and Lagacé (2021) highlight that the way older adults experience and acknowledge and react to age-based discrimination and ageist stereotypes may also be culturally dependent. The cultural perpetuations of ageism and how older adults are viewed in the country as reflected in the lyrics of various calypso or Soca music (styles of music originated in Trinidad & Tobago). The first verse of a calypso, “He No Dead Yet” sung by King Fighter (birth name: Shurland Wilson) in 1962 speaks about the family of an older man making plans for his assets while he is still alive but sick in bed, not dead yet. In a 2023 Soca release by Crazy (birth name: Edwin Ayoung) titled “Too Old To Soca” he talks about people scoffing at him and telling him that he should be in bed, instead of being in parties or on a stage performing, because of his age.

In many studies, including Goll (2015), and Holt-Lunstad (2018), emphasis has been placed on loneliness among older adults being a major public health issue and the possible association with processes of social participation and identity. However, none of the participants in our study – regardless of their living arrangements - expressed experiences of loneliness, lack of emotional support or social isolation. This could also be attributed to a cultural phenomenon due to common family structures within Trinidad & Tobago with close-knit, compound families and extended family households. A cross-sectional survey conducted in Granada, southern Spain found that perceived health and perceived social support predicted life satisfaction (Dumitrache et al., 2017). This finding supports the results from our study as participants had no complaints about the social/emotional support provided to them, had good self-reported health status and were generally satisfied with life at this stage.

A qualitative study conducted across six Caribbean countries revealed disparities in access to healthcare and social services, influenced by both socio-economic status and geographic location (Cloos et al., 2010). The study also highlighted differences in family support, concerns about social isolation, varying levels of social engagement, and insufficient social protection benefits, particularly regarding income. Cloos recommended adopting a comprehensive, multi-sectoral strategy based on the ‘active ageing’ framework to promote healthy ageing throughout the region.

The coping strategies mentioned by participants that were used in dealing with other ageing-related challenges were considered to be healthy or proactive. Social support or socialization, maintaining physical and mental health through various activities, and spirituality/religion were fundamental to older adults’ coping. This was much aligned to a study which showed that leisure activities, such as voluntary work and hobbies explain a significant part of older adults’ social connectedness (Toepoel, 2013). Another study conducted in the UK showed that religion, spirituality and/or belief was a source of strength, comfort and hope in difficult times and brought about a sense of community and belonging in the everyday lives of the older adults (Malone & Dadswell, 2018). According to Zimmer et al. (2016) there is evidence that religiosity is associated with longer life and better physical and mental health. When looking at other health outcomes, Zimmer also found a statistical correlation between religiosity, spirituality, and health.

Mamen (2018) presents positive ageing as the process of maintaining a positive attitude, feeling good about yourself, keeping fit and healthy, and engaging fully in life as you age. Based on this definition, most of the participants in this sample were positively ageing. Most people found joy in the ageing experience; they did not feel their age – some felt the same as they had in their younger days. The main thing was that while participants were personally enjoying ageing, society’s perceptions of ageing and preconceived notions of what older adults should look, act, and be like, affected them. The findings of our study, as it relates to how older adults feel about and view ageing, show the relevance of continued societal participation among older adults for a healthy ageing society. “Ability to contribute to society” is listed as one of the five functional ability domains required for healthy ageing by the UN Decade of Healthy Ageing (Ahlawat, 2023). Not only can it contribute to developing strong relationships and a sense of purpose among older adults, but it also can provide benefits to the country’s economy. An example of this would be older adults’ workforce opportunities. The Government proposing to increase the retirement age from the current 60 to 65 years of age, giving employees the option to work for five more years before accessing their Retirement Benefit, is mutually beneficial to older adults and the country.

The findings reveal that older adults’ positive outlook on ageing, confidence in their health, adaptability to modern changes, and deliberate mental preparation reflect core principles of both the General Ecological Model of Ageing and the WHO Active Ageing Framework. These themes emphasize the individual level of the ecological model where beliefs, resilience, coping strategies, and personal health behaviors shape one’s ageing trajectory while also highlighting the influence of social and environmental systems, such as family support, cultural norms, community safety, healthcare experiences, and access to social services. Similarly, the Active Ageing Framework’s pillars of health, participation, and security are evident as participants maintain physical and mental wellbeing, stay engaged socially and spiritually, and strive for financial independence and safety despite challenges like ageism, crime fears, and systemic barriers in health and social services. Together, these interconnected results illustrate that positive personal mindsets, supportive environments, and opportunities for engagement work synergistically to create a more empowered and fulfilling ageing experience, aligning strongly with both frameworks’ emphasis on holistic, dynamic, and context-driven ageing.

While some findings may resonate with a wider regional spectrum such as the perceptions towards ageing, health and social connectedness, others may be context-specific to Trinidad, they can serve as models for other Caribbean nations. Quashie et al. (2018) stated that the Caribbean region is experiencing increasingly rapid population ageing, with the proportion of individuals aged 60 and over expected to rise from 14% in 2015 to 25% by 2050. According to the report, in 2002, the Madrid International Plan of Action on Ageing (MIPAA) was adopted, prompting Caribbean nations to develop national ageing policies and enhance programs and services for older adults in areas such as health, pensions, and caregiving. Despite these positive steps, many countries continue to face major gaps between policy and practical implementation, largely due to limited financial resources, political inaction, and inadequate infrastructure. While Caribbean nations vary in many respects, they share common cultural values, and the challenges associated with an ageing population such as health care inequalities and inefficiencies represent a wider regional reality (Quashie et al., 2018).

### Recommendations and Future Research

Findings from our study have implications for older adults and their caregivers, as well as for practice and directions for future research. Locally, in Trinidad & Tobago, there needs to be a change in the framing of age and ageing at both the individual and societal levels with the promotion of healthy ageing through interventions that increase positive views of ageing and improve health. A recommendation to address this is to initiate consistent, sustainable nationwide community campaigns and media initiatives to change the narrative around age and ageing. Better accommodations and more assistance and can be provided by governmental, non-governmental, and corporate entities to facilitate and prepare the population for the inevitable ageing process, specifically as it relates to restroom facilities, seating areas and education on retirement, pension, and socialization.

Even when considering age-related health issues, there is a lack of geriatric facilities and specialized clinics in regional health systems, and some health care professionals lack knowledge and awareness to be able to provide safe, timely and quality care. In lieu of that infrastructure, and to close that knowledge gap, another recommendation is that healthcare professionals should have mandatory training within their programs to educate them on gerontology competencies - how to understand and deal with implicit biases regarding ageing. The continued education will also assist in allowing these professionals to better understand older persons and to exhibit professional levels of empathy and sensitivity. These educational and sensitization sessions should be incentivized for attendance, conducted by qualified persons and should engage an interdisciplinary team such as local gerontologists and psychologists to increase awareness and address the cultural and subconscious implications of ageism.

To ensure that older adults locally continue to maintain healthy social interactions, more effort can be made to maintain the existing senior citizen activity centers with regular scheduled educational and recreational activities tailored to healthy ageing, and an adequate quota of trained staff and volunteers. There is also opportunity to ensure that all communities (rural and urban) have access to these centers, whether publicly or privately run as this could be a very beneficial public service for the ‘sandwich generation’ (those caring for older parents and young children simultaneously) and can be used to promote intergenerational socialization.

Additionally, there is an opportunity for more studies on reframing ageing at the individual, institutional and societal level. Further research could also examine undesirable cultural norms such as undesired or ageist labels as it relates to this population, and further self-perceptions of ageing using a larger demographic range to determine how perceptions differ at different ages or based on gender. Multilevel and multisector efforts are required to reduce everyday ageism and promote positive beliefs, practices, and policies related to aging and older adults.

While a National Policy on Ageing exists for Trinidad and Tobago, the Caribbean Public Health Agency states that there is a need for the development of evidence-based practices and policies from the experiences of older persons (Caribbean Public Health Agency, 2020). This suggests a lack of enforcement mechanisms for this act, which was in revision at that time, and continues to be to date. The main policy recommendation from this study would be prioritize the revision and institution of this legislative mandate and to launch a National Plan of Action for Ageing with measurable targets, governmental coordination, and civil society engagement. The insights from this study can also contribute to a broader Caribbean discourse on population ageing, highlighting the need for multi sectoral respond, cultural mindset shifts and the potential for cross-country collaboration on solutions.

### Limitations and Strengths

Strengths of this study were in its design which provided rich insights into the ageing experience in Trinidad & Tobago while preserving the voice and firsthand perspective of participants. The study highlighted various perceptions of ageing while also considering the assumptions and provided practical applications and supporting evidence for the work needed to be done for an improved environment for older adults. A main limitation of the study was the in-person data collection. As a result, older adults who were immobile or unwell were excluded from the study. The recruitment flyer for the focus groups was also shared predominantly online, which excluded many sub-populations of the target sample. Other limitations to consider include lack of further demographic segmentation such as ethnic, religious, socioeconomic variation, and limited gendered patterns.

## Conclusion

In conclusion, this study highlighted the perceptions of ageing of older adults in Trinidad and Tobago. It identified their perceptions of ageing and health, some of their lived experiences, their perceptions of health and social services offered, and their mechanisms of adaptation and self-regulation when faced with challenges. Gaining a better understanding of the individual perceptions of these older adults allows the opportunity to respond to the challenges or issues that may exist during this experience and to ensure that their environment provides the necessary resources to improve and maintain their physical health, mental health, and overall well-being. With the significant change in the global population as it relates to ageing, this paper provides insight on the way older adults of Trinidad and Tobago perceive ageing, as we work towards a healthier and happier ageing population.

## Data Availability

Data available on request from the authors.
